# Subclinical Hypothyroidism among local adult obese population

**DOI:** 10.12669/pjms.344.14127

**Published:** 2018

**Authors:** Abdul Sami, Malik Faisal Iftekhar, Muhammad Abdur Rauf, Akhter Sher

**Affiliations:** 1 Dr. Abdul Sami, FCPS. Department of Cardiology, Hayatabad Medical Complex, Peshawar, Pakistan; 2 Dr. Malik Faisal Iftekhar, FCPS. Department of Cardiology, Hayatabad Medical Complex, Peshawar, Pakistan; 3 Dr. Muhammad Abdur Rauf, FCPS. Department of Cardiology, Hayatabad Medical Complex, Peshawar, Pakistan; 4 Dr. Akhter Sher, FCPS, Department of Cardiology, District Headquarter Hospital, Swabi, Pakistan

**Keywords:** Body mass index, Hypothyroidism, Obesity, Thyroid stimulating hormone, Thyroxine

## Abstract

**Objective:**

To determine the frequency of subclinical hypothyroidism in local adult obese population.

**Methods:**

The study was conducted at Hayatabad Medical Complex, Peshawar, from March, 2017 to August, 2017. All patients aged between 18 and 60 years with BMI of more than 29kg/m^2^ were included in the study. Patients on lipid lowering drugs, with renal failure, hepatic failure and already diagnosed cases of thyroid dysfunction were excluded from the study. Thyroid functions were measured for all patients.

**Results:**

A total of 127 adults were included in the study in a consecutive manner. Mean age was 34.5 + 7.9 years of which 46.5% were male and 53.5% were female. Mean BMI was 32.05±2.06 kg/m^2^. The mean serum TSH was 3.13±1.10 mIU/L and mean serum thyroxine level was 1.08±0.25ng/dl. Subclinical hypothyroidism was recorded in 15% of the study population.

**Conclusion:**

Subclinical hypothyroidism is highly prevalent in our population with BMI of more than 29kg/m^2^. Further studies are recommended on relationship between thyroid functions and BMI and its effect on cardiovascular functions.

## INTRODUCTION

Obesity, as defined by the BMI, is on the rise globally and in most sex-age groups is more than 30% in United States. Obesity prevalence has not altered much over past 12 years from 1999 to 2010. This trend in obesity prevalence is linear for men while it is not significant for women over this period.^1^

The three major non-communicable diseases (NCDs) with highest mortality rate in Pakistan are cardiovascular diseases; malignancies and chronic respiratory diseases.^2^ All these NCDs have few common risk factors like smoking, alcohol intake, diabetes mellitus, hypertension, dyslipidemia, obesity, unhealthy & sedentary lifestyle^3^; which can be changed resulting in prevention of these NCDs. Besides, studies have shown Asians to have a higher BMI than Europeans.^4^ Therefore, a lower cutoff value of BMI for overweight (23.0–24.9 kg/m2) and obese (>25.0 kg/m2) Asians has been suggested by the authorities of the international obesity task force.^4^ WHO figures reveal increasing prevalence of overweight and obese adults.^5^

Growing evidence suggests association of altered thyroid function and obesity, a lasting state of low grade inflammation. Recent data has revealed leptin, an adipocyte hormone, to be a major factor linking obesity and thyroid autoimmunity.^6^ Recently, several clinical studies have evaluated the issue of hormonal changes associated with obesity.^7^

The prevalence of subclinical hypothyroidism in Pakistani population is 5.4% compared to the prevalence of overt hypothyroidism that is 4.1% and compared to males, females are more affected.^8^ There are studies that reveal slightly elevated thyroid stimulating hormone level within reference range relating to slightly increased body weight.^8^-^10^ It has been reported that 100-fold changes in thyroid stimulating hormone can be caused by only two-fold changes in free thyroxine level and that a unit increase in thyroid stimulating hormone level can cause a 3% increase in the components of metabolic syndrome.^11^,^12^

The present study was designed to determine the frequency of subclinical hypothyroidism in local adult obese population. Doing a thorough literature search, we found that very little data exists about the thyroid hormone levels and its dysfunction among local adult obese population. This study will be an attempt to determine the frequency of subclinical hypothyroidism in local obese adult population and the results of this study will be a guideline for devising future research strategies and identifying mechanisms of preventing subclinical hypothyroidism in patients with morbid obesity.

## METHODS

This descriptive cross-sectional study was conducted at Hayatabad Medical Complex, Peshawar from March 2017 to August 2017. It was a. All patients aged between 18 and 60 years with BMI of more than 29kg/m^2^ were included in the study. Patients on lipid lowering drugs, with renal failure, hepatic failure and already diagnosed cases of thyroid dysfunction were excluded.

All subjects were subjected to complete history and clinical examination to exclude effect modifiers. From all the patients, a blood sample was obtained and was sent to hospital laboratory to measure the serum TSH & Thyroxine levels (this was done free of cost from hospital laboratory). All the above-mentioned information including name, age, and sex was recorded in a pre-designed proforma and strict exclusion criteria was followed to control confounders and bias in the study results. All the investigations were done form hospital laboratory by single experienced microbiologist having minimum of five years of experience.

Data was stored and analyzed in SPSS version 20. Mean and standard deviation (SD) were calculated for quantitative variables like age, serum TSH and thyroxine levels. Frequencies and percentages were calculated for categorical variables like gender and subclinical hypothyroidism. Subclinical hypothyroidism was stratified among age and gender to see the effect modifications. Chi square was used and a value less than 0.05 were considered significant.

## RESULTS

Total of 127 adult participants having a body mass index of more than 29kg/m^2^ were observed to determine the frequency of subclinical hypothyroidism and results were analyzed.Age distribution among 127 patients was analyzed as; 44(34.6%) patients were <30 years, 45(35.6%) patients were in age range 30-40 years, 38(29.9%) patients were in age range above 40 years. Mean age of the study population was 34.5 ± 7.9 years. Out of 127 patients included in the study, there were 46.5% male and 53.5% female . Mean BMI was 32.05 ± 2.06. BMI was stratified into two classes i.e. BMI between 29.5-32.0kg/m^2^ and BMI between 32.1-35.5kg/m^2^.

All the patients were subjected to measurement of serum TSH and thyroxine levels. The mean serum TSH was 3.13 ± 1.10 mIU/L and mean serum thyroxine level was 1.08 ± 0.25ng/dl. Subclinical hypothyroidism was recorded in 15% of study population. Subclinical hypothyroidism was stratified with regards to age groups, gender and BMI categories.

**Table-I:** Summary of Patient Characteristics (n =127).

Characteristics	Subclinical Hypothyroidism		p-value

	Present	Not present	
***Age***			0.001
<30 years	0	44	
30-40 years	13	32	
>40 years	6	32
***Gender***			0.159
Male	6	53	
Female	13	55
***BMI***			0.103
29.0-32.0 kg/m^2^	6	56	
32.1-35.5	13	52	

## DISCUSSION

Rising weight gain and obesity is rapidly becoming a grave problem globally^13^ due to its well-established association with diabetes^14^,^15^, hypertension^15^, heart diseases^15^, ischemic stroke^16^, and several types of malignancies.^17^ On one hand, sedentary lifestyle leading to a disparity between consumption and utilization of energy causes weight gain while on other hand, thyroid dysfunction leading to impaired resting energy expenditure (REE) balance is an established entity leading to weight gain and obesity.^18^-^20^

In our study, the mean BMI was 32.05 ± 2.06 with mean TSH was 3.13 ± 1.10 mIU/L and mean serum thyroxine level was 1.08 ± 0.25ng/dl. Hypothyroidism was recorded in 15% of the patients in our study. Like these findings, Michalaki et al.^21^ also found low occurrence of thyroid impairment in obese patients with elevated TSH. Likely, in NHANES III survey, occurrence of thyroid antibodies was similar in patients who were morbidly obese with normal TSH and in general population.^22^ There is positive association between FT3 to FT4 ratio and BMI while serum TSH also rises with increasing collection of fat cells. As suggested by Biondi^23^, the increased deiodinase activity because of increased fat collection leads to raised conversion of T4 to T3 which compensates for a better energy utilization.

The aberrantly raised TSH levels in obese patients regress after weight loss either by lifestyle modification or surgical intervention.^20^ This finding suggests that autoimmune destruction of thyroid cells was not the reason for elevated serum TSH levels. Studies failed to demonstrate an association between thyroid hormone levels and BMI despite a suggestion that altered thyroid function in turn leads to obesity.^24^ Lately, studies on rats being fed high fat diet revealed a rise in serum TSH levels despite normal levels of FT4and FT3.^25^

Thus, by summing up the past studies, two different theories arise showing relationship of thyroid function and obesity:

1. Lack of thyroid hormone leads to weight gain culminating in overt obesity, which in turn predisposes patients to develop autoimmune hypothyroidism.
2. Sometimes, raised TSH in obese patients does not show hypothyroidism but it is the result of weight gain rather than its cause.

## CONCLUSION

Subclinical hypothyroidism is highly prevalent in our population with BMI of more than 29kg/m^2^. Further studies are recommended on relationship between thyroid functions and BMI and its effect on cardiovascular functions.

### Author`s Contribution

**Abdul Sami** conceived, designed and did statistical analysis & manuscript writing.

**Malik Faisal Iftekhar & Muhammad Abdur Rauf** did data collection and editing of manuscript.

**Akhter Sher** did review and final approval of manuscript.
